# Exploring Associations between Problematic Internet Use, Depressive Symptoms and Sleep Disturbance among Southern Chinese Adolescents

**DOI:** 10.3390/ijerph13030313

**Published:** 2016-03-14

**Authors:** Yafei Tan, Ying Chen, Yaogui Lu, Liping Li

**Affiliations:** Center for Injury Prevention Research, Shantou University Medical College, Shantou 515041, China; tanyf1012@163.com (Y.T.); 13chenying@stu.edu.cn (Y.C.); laoyaoguai6@163.com (Y.L.)

**Keywords:** adolescents, problematic Internet use, depression, sleep disturbance

## Abstract

The primary aim of this study was to examine associations between problematic Internet use, depression and sleep disturbance, and explore whether there were differential effects of problematic Internet use and depression on sleep disturbance. A total of 1772 adolescents who participated in the Shantou Adolescent Mental Health Survey were recruited in 2012 in Shantou, China. The Chinese version of the Internet Addiction Test (IAT) was used to evaluate the prevalence and severity of Internet addiction. The Chinese version of the Pittsburgh Sleep Quality Index (PSQI), a 10-item version of the Center for Epidemiologic Studies Depression Scale (CESD-10), and other socio-demographic measures were also completed. Multiple regression analysis was used to test the mediating effect of problematic Internet use and depression on sleep disturbance. Among the participants, 17.2% of adolescents met the criteria for problematic Internet use, 40.0% were also classified as suffering from sleep disturbance, and 54.4% of students had depressive symptoms. Problematic Internet use was significantly associated with depressive symptoms and sleep disturbance. The correlation between depressive symptoms and sleep disturbance was highly significant. Both problematic Internet use (*β* = 0.014; Sobel test *Z* = 12.7, *p* < 0.001) and depression (*β* = 0.232; Sobel test *Z* = 3.39, *p* < 0.001) had partially mediating effects on sleep disturbance and depression was of greater importance for sleep disturbance than problematic Internet use. There is a high prevalence of problematic Internet use, depression and sleep disturbance among high school students in southern China, and problematic Internet use and depressive symptoms are strongly associated with sleep disturbance. This study provides evidence that problematic Internet use and depression have partially mediating effects on sleep disturbance. These results are important for clinicians and policy makers with useful information for prevention and intervention efforts.

## 1. Introduction

Over the past ten years, the popularity of Internet use among adolescents has dramatically increased; 93% of teens between the ages 12–17 go online in the U.S., as do 93% of Japanese adolescents [1]. China had 256 million adolescent Internet users as of January 2014, accounting for 71.8% of the overall number of adolescents [2]. Given the swift increase in numbers of teenage users, it is not surprising that the question has been raised of whether the advantages of adolescent Internet use outweigh the disadvantages. Internet-based programs can provide opportunities for interactivity and active participation that might not otherwise be available to adolescents. There is evidence that computer game practice improves the spatial performance and iconic and visual attention skills of adolescents [3]. However, excessive Internet use may lead to grey matter atrophy in the brain [4], negatively affecting concentration and memory, as well as the ability to make decisions and set goals [5]. In addition, heavy Internet use may cause psychological disorders, such as Internet addiction, depression, and anxiety [6].

Studies have found that excessive Internet use is strongly associated with depressive symptoms, and that adolescents involved with problematic Internet use are vulnerable to psychological disorders, such as depression [7,8,9,10]. Conversely, depression in adolescents are more likely to lead to Internet use problems [11], which is also supported by Brunet’s longitudinal study [12]. However, the nature and causal directionality of the relationship between problematic Internet use and depression are unclear.

Internet addiction and other problematic Internet use behaviors may have significant influence on the sleep-wake schedule, leading to insomnia and other sleep disturbances [13]. In previous studies, heavy Internet use was found to be associated with insomnia [14], and increased time spent on the Internet led to the significant disturbance of sleep [15]. Other previous studies have found evidence that time spent on digital game-playing and problematic Internet use are associated with sleep delays, irregular sleeping patterns and excessive daytime sleepiness [15,16,17], which in turn are associated with increased waking-time tiredness [18]. One psychophysical mechanism that may help to explain the negative impact of problematic Internet use on sleeping habits may be that night-time computer usage leads to a state of high arousal, thus interfering with the calming processes that are necessary for sleep [19].

Sleep disturbances are highly interrelated with a depressive status [20,21,22]. To be diagnosed with major depression, an individual must have at least five of nine criteria for depressive symptoms, and disordered sleep is one of the nine symptoms. More than two-thirds of depressed children and adolescents suffer sleep-onset or sleep-maintenance problems [20,23], even though the manifestation of sleep disturbance is not necessary for a diagnosis of major depression. Researchers have suggested that the relationship between sleep disturbance and depression is bidirectional [24], and that the two conditions could feed back on each other to mutually maintain their existence [25]. Longitudinal research has demonstrated that sleep disturbance is associated with an increased risk of developing depression [26,27], and adolescents reporting sleep disturbances may display depressive symptoms within one year [28]. Depression may also lead to sleep disturbance in depressed children by way of disturbing circadian regulation, maintaining a negative state, and reducing both regular exposure to bright light and social activities [29,30].

To date, despite the relationships among problematic Internet use, depression and sleep disturbance have been explored by numerous studies, relatively few studies have examined whether problematic Internet use and depression have differential effects on sleep disturbances among adolescents. Hence, we conducted a cross-sectional study to fill the gap and explore the associations between problematic Internet use, depression and their differential effects on sleep disturbance. We predicted that: (1) problematic Internet use would be associated with higher levels of depression and sleep disturbance, and depression would be associated with sleep disturbance; and (2) problematic Internet use and depression would have differential effects on sleep disturbance.

## 2. Methods

### 2.1. Sample

The Shantou Adolescent Mental Health Survey was a cross-sectional survey conducted in 2012 in Shantou, China. Three high schools, each representing a different type of school (city-level magnet school, provincial/regional-level magnet school, and technical school) were included in the study. Twenty six classes were randomly selected to participate in the survey, including eight 7th grade classes, nine 8th grade classes, and nine 9th grade classes. A total of 1727 students were invited to participate in the investigation, and 1661 students completed the entire questionnaire, resulting in a 96.2% response rate. All available students were fully informed of the purpose of the investigation and participated voluntarily. All participants and their parents or guardians approved of the study and voluntarily signed written consent letters, and this study was approved by the Ethics Committee of the Medical College of Shantou University (No. SUMC2012XM-0070).

### 2.2. Measures

#### 2.2.1. Depression

The Center for Epidemiologic Studies Depression Scale (CESD)-10 was used as a depression screening scale [31]. The response options were in four categories ranging from ”rarely” (0) to ”all of the time” (3). Two items in the scale required reverse coding. All items were summed into a total score that ranged from 0 (no depressive symptoms) to 30 (severe depressive symptoms). According to Li’s criterion [31], we classified a CESD-10 score >8 as depression. In the present survey, the Cronbach’s alpha of the Chinese CESD-10 was 0.84.

#### 2.2.2. Sleep Disorders

The Pittsburgh Sleep Quality Index (PSQI) was used to assess Chinese adolescents’ sleep disturbance [32]. The PSQI evaluates sleep quality and disturbance over a 1-month period. Nineteen individual items generated seven component scores, which were summed to produce a global score with a range of 0 to 21. Higher scores represented poorer subjective sleep quality. In line with Xu’s standard [33], sleep disturbance was classified as a PSQI score >5. In the current study, the Cronbach’s alpha of the PSQI for the Chinese version was 0.83.

#### 2.2.3. Internet Use and Problematic Internet Use

##### Duration of Internet Use

All participants were asked the following question: “During the past month, how many days did you use the Internet each week?” and “During the past month, on average, how long did you use Internet every day?” We multiplied the days of Internet use and hours per day to get the Internet use hours per week.

##### The Impact of Internet Use

The participants were also asked questions about the effect of their Internet use on their lives, including its effects on their health, school performance and family relationships. The response options for each question included “very negative”, “negative”, “no effect”, “positive” and “very positive”. A dichotomous variable indicating negative (*i.e.*, “very negative” or “negative”) effects was created for each of the three domains.

##### Problematic Internet Use

The Internet Addiction Test (IAT) was used to determine the severity of Internet addiction [34]. The test contained 20 self-reported items each rated on a scale from 1 to 5, where a score of 1 was defined as “rarely” and 5 as “always”, and included how adolescent Internet behavior affected their daily lives, social intercourse, sleeping patterns and feelings. The total score for each participant could range from 20 to 100, and high scores indicated greater problems associated with Internet use. According to Lai’s criterion [34], a score of 20 to 49 indicated average Internet use, 50 to 79 represented problematic Internet use, and over 79 scores represented Internet dependence. In this study, we used a cut-off score of 50 to define the problematic Internet use. In this present survey, the Cronbach’s alpha for IAT was 0.90.

#### 2.2.4. Socio-Demographic and Family Factors

Other measures used in the study’s analyses included the student’s gender, school grade, annual household income, parents’ highest level of education, course scores and whether or not the child was living in an intact family.

### 2.3. Statistical Analysis

Descriptive statistics were generated for the main variables examined in the study, including IAT, PSQI and CESD scores. A bivariate analysis was subsequently conducted, examining the relationships of problematic Internet use with sleep disturbance and depressive symptoms, as well as the associations between the demographic variables and each of the three key variables. Correlation analysis was used when both variables were continuous, a *t*-test was used when one variable was a dummy variable and the other was continuous, and ANOVA was used when one variable was categorical with more than two categories, and the other one was continuous. Path-analytic mediation analyses were utilized to determine the relationship between problematic Internet use, depression and sleeping disturbance in line with the method proposed by Baron and Kenny [35]. To establish mediation regression models, the following criteria had to be met:

(1) The independent variable must significantly account for dependent variable.
(2) The independent variable must significantly predict the mediator variable.
(3) The mediator variable must account for the dependent variable.
(4) When the mediator variable is controlled, the relationship between the independent variables and the dependent variable should be decreased or is no longer significant.

A perfect mediational model is established if the association between the independent variables and the dependent variable is reduced to zero. The Sobel test [36] was used to determine whether the mediator variable significantly influenced independent variables to the dependent variable. Significance was assessed based on a two-tailed test with a critical value set at 0.05. All analyses were conducted in SPSS version 21.0. (SPSS Inc.: Chicago, IL, USA).

## 3. Results and Discussions

Among the 1661 high school students, 51.8% were male and 48.2% were female, and the average age was 14.53, with a range of 12 to 18 years, and 96.9% of sample ranging from 13 to 16 years. The mean number of hours of Internet use per week was 6.21 h. About 18% of the students went online almost every day, and 9.6% of the students usually used the Internet for more than 5 h at a time. The mean IAT score was 36.91, with 17.2% of students being categorized as engaging in problematic Internet use. The mean CESD score was 9.69, with 54.4% of students identified as having depression symptoms, and the mean PSQI score was 5.16, with 40.0% of the students being classified as having sleep disturbance (See Table 1).

Table 2 shows the correlations between problematic Internet use and the two main outcome variables, depressive symptoms and sleep disturbance. The IAT score was significantly associated with depressive symptoms and sleep disturbance. The correlation between depressive symptoms and sleep disturbance was highly and statistically significant.

The associations of problematic Internet use, depressive symptoms and sleep disturbance with gender, school performance, family factors and reported negative effects of Internet use are reported in Table 3. The *t*-test analyses found the reported negative effects of Internet use on health, school performance and family relationships to be individually associated with IAT, PSQI and CESD scores. For males, lower school scores, coming from a non-intact family and high level of father’s education were associated with problematic Internet use. For females, high school grade level, lower school scores, coming from a non-intact family and low level of father’s education were factors associated with higher CESD scores. For females, high school grade, coming from a non-intact family, low level of father’s education and high level of mother’s education were factors associated with higher PSQI scores.

**Figure 1:**
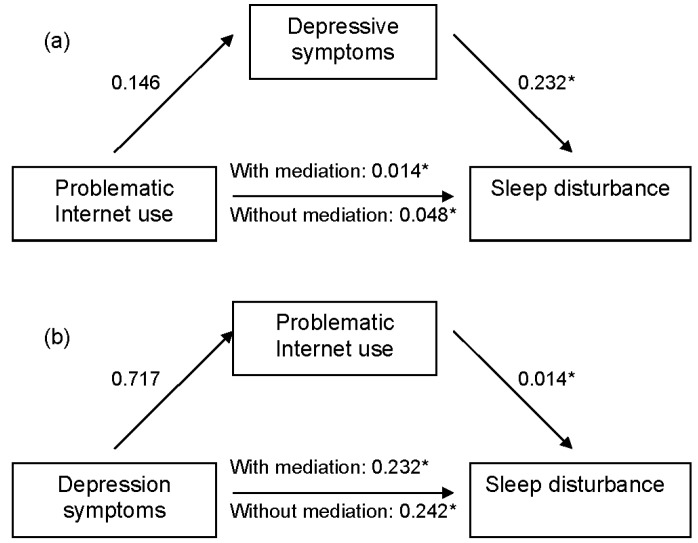
Mediation analyses of relationship between problematic Internet use, depressive symptoms, and sleep disturbance. (**a**) Depressive symptoms as a mediator variable between problematic Internet use and sleep disturbance; (**b**) Problematic Internet use as a mediator variable between depressive symptoms and sleep disturbance; * *p* < 0.001.

As Table 4 shows, model I presents the mediational model of depressive symptoms as the mediator between problematic Internet use and sleep disturbance (Figure 1a). Problematic Internet use was found to be a significant predictor of sleep disturbance (*β* = 0.048, *p* < 0.001) and depressive symptoms (*β* = 0.146, *p* < 0.001), and depressive symptoms significantly predicted sleep disturbance (*β* = 0.08, *p* < 0.001). When the mediator (depressive symptoms) was controlled, problematic Internet use also significantly predicted sleep disturbance (*β* = 0.014, *p* = 0.001). This suggested that depression was a partial mediator between problematic Internet use and sleep disturbance, with the mediator responsible for 70.6% (0.146 × 0.232/0.048) of the whole effect. The Sobel test confirmed that depression was a significant mediator (*Z* = 12.70, *p* < 0.001). In model II, problematic Internet use was a mediator between depression and sleep disturbance (Figure 1b). Depression was associated significantly with sleep disturbance (*β* = 0.242, *p* < 0.001) and problematic Internet use (*β* = 0.717, *p* < 0.001), and problematic Internet use was associated significantly with sleep disturbance (*β* = 0.014, *p* = 0.001). When problematic Internet use was taken into account, depression was significantly associated with sleep disturbance (*β* = 0.232, *p* < 0.001), and problematic Internet use was a partial mediator accounting for 4.1% (0.717 × 0.014/0.242) the whole effect. The Sobel test indicated that the problematic Internet use was a significant mediator (*Z* = 3.39, *p* < 0.001).

There have been rapid increases in Internet use in society in recent years, and the potentially harmful effects of excessive Internet use on the health of individuals, and of youth in particular, have been described by many studies. However, the effects of problematic Internet use and depression on sleep disturbance are not well understood. To our knowledge, this study is the first to begin to elucidate the inter-relationship of problematic Internet use and depression on the effects of sleep disturbance among Chinese adolescents, and its underlying mechanisms.

In our study, the prevalence of problematic Internet use was 17.2% among adolescents, with 40.0% of adolescents suffering from sleep disturbance, and up to 51.4% of adolescents had depressive symptoms. These results were highly similar to a study in Hong Kong, China [37]. The prevalences in our sample were higher than those reported in previous studies [38,39,40]; it might be possible that we adopted different methods and criteria in data collection, and moreover, adolescents were in a special growth phase, mentally and physically unstable and undergoing great changes.

Although the Internet positively affects our daily lives, our findings suggest that problematic Internet use had negative effects on the physical health, academic performance and family relationships of adolescents, similar to a study in Taiwan [41], which found that the Internet negatively influenced many aspects of student’s lives. Adolescents who were problematic Internet users perceived significantly stronger negative Internet influence on daily routines, school performance, and parental relationships than the non-dependents. These results showed the importance of proper supervision and monitoring of a child’s Internet use by parents and teachers.

With regard to the relationship between problematic Internet use, depressive symptoms and sleep disturbance, the current study found that problematic Internet use was positively associated with the level of depressive symptoms and sleep disturbance, as indicated by bivariate analyses, which supported our first prediction. The total scores on the Internet addiction test were correlated strongly with both the depressive symptom scores and sleep disturbance. Additionally, depressive symptoms were highly associated with sleep disturbance. A prior study showed that moderate/high Internet addiction might be applied to deal with depressive symptoms, but excessive Internet use could lead to further depressive symptoms [42]. Depressive adolescents also suffer more easily from problematic Internet use [11]. In addition, students with problematic Internet use may hide their vulnerable and negative feelings, such as depression, to escape from the negative or stressful events caused by excessive Internet use [43]. In the same way, previous reports indicate that Internet addiction has a close association with sleep disturbance [15,44]. Their results show that the prevalence of sleep problems is higher among Internet-addicted students, and excessive Internet use could affect our health indirectly through lack of sleep. The relation between depression and sleep disturbance is consistent with previous studies findings that depression is the main factor affecting sleep, and insomnia could increase the risk of depression and also reflect the depressive symptoms [21,44].

Our study also extended previous research by revealing the relationships between problematic Internet use, depression and sleep disturbance, showing that problematic Internet use and depression had differential effects on sleep disturbance, which supports prediction 2. In model I, we found that when depression was a mediator, problematic Internet use had negative effects on sleep disturbance, and depression partially mediated the association between problematic Internet use and sleep disturbance, with the mediator responsible for 70.6% of the whole effect. That is to say, there was about 70.6% of the effect of problematic Internet use on sleep disturbance going though depression indirectly and the direct effect of problematic Internet use on sleep disturbance was 29.4%. In model II, depression and sleep disturbance were partially mediated by problematic Internet use, and the mediating effect of problematic internet use was responsible for 4.1% of whole effect, indicating that approximately 95.9% of the effect of depression on sleep disturbance was direct. Comparison of the results of two models showed that depression exerted a stronger mediating effect than problematic Internet use, and also indicated that depression was closely associated with sleep disturbance. These suggested that depression would play a more important role than problematic Internet use when considered as a mediator. Sobel tests of the two models showed that the *Z*-score in model I in which depression was a mediator was higher than in model II where problematic Internet use as a mediator, which meant that the accuracy of model I was higher than model II, further confirming the conclusion that depression exerted a stronger mediating effect than problematic Internet use. Therefore, we concluded that problematic Internet use and depression partially mediated the relationship of sleep disturbance with these two variables, and depression as a mediator exerted stronger effect for sleep disturbance than problematic Internet use.

Several limitations should be noted with regard to this study. First, because it was a cross-sectional study, our results were not able clearly to indicate causal directionality, *i.e.*, we could not say whether problematic Internet use or depressive symptoms precede the development of sleep disturbance. Longitudinal research is needed to address this problem, and help us better understand the inter-relationship and underlying mechanisms connecting Internet overuse and depressive symptoms with sleep quality. Second, the data were reported by the students themselves, which might result in self-report bias and even undermine authenticity of data, though our investigators emphasized that the questionnaires must be finished truthfully. Third, although the standardized scales were adopted as screening tools to assess psychological problems and poor sleep quality, these measurements could not be equivalent to clinical diagnoses, therefore, more clinical diagnosis by qualified psychiatrists are needed in future studies. Fourth, other factors, such as urbancity or race, were been taken into consideration in our study. Students who are from different areas and races may have different individual chacteristics. In addition, our measurement of Internet use also had its limitations, as it did not specify the time of day of Internet use. It is likely that Internet use at night is more detrimental to the patterns and quality of adolescent sleep than daytime Internet use, as discussed above. Finally, the subjects recruited only from the junior high schools in a southern Chinese city, so one should be cautious in generalizing our findings to Chinese adolescents.

## 4. Conclusions

The current study demonstrated the high prevalence of problematic Internet use, depression and sleep disturbances among Chinese adolescents, and shed light on the fact that problematic Internet use is associated with depression and sleep disturbance and depression is associated with sleep disturbance. This study provided new information indicating that problematic Internet use and depression have partially mediating effects on sleep disturbance. We hope our study provides clinicians and policy makers with useful information for prevention and intervention efforts.

## Figures and Tables

**Table 1:** Distribution of socio-demographic, Internet use, depressive symptoms and sleep disturbances (*N* = 1661).

Variables	*N*/Mean	%(*N*)/SD
**Socio-Demographic Factors**		
**Gender**		
Male	860	51.8
Female	801	48.2
**School Grade**		
7th grade	522	31.4
8th grade	599	36.1
9th grade	540	32.5
**Father’s Education Background**		
Primary school or less	476	28.7
High school	768	46.2
University or more	417	25.1
**Mother’s Education Background**		
Primary school or less	668	40.2
High school	872	52.5
University or more	121	7.3
**Family Income (per Year)**		
<CNY 20,000	480	31.7
CNY 20,000—69,999	643	42.5
≥CNY 70,000	390	25.8
**Intact Family**		
No	114	6.9
Yes	1534	93.1
**Single-Child Family**		
No	908	56.6
Yes	695	43.4
**Most Courses in the Last Report Card**		
80–100 or A’s	755	55.1
60–79 or C’s	494	36.1
Below 60 or F’s	121	8.8
**Internet Use Factors**		
**Days using Internet per Week**		
0–1	496	30.0
2–6	859	52.0
Almost everyday	297	18.0
**Hours of Using Internet per Time**		
<2 h	922	58.2
2–5 h	472	29.8
>5 h	190	12.0
**Internet Use Hours per Week**	6.21	6.88
**Internet Addition Test score**	36.91	13.66
Non-problematic Internet use	1373	82.8
Problematic Internet use	285	17.2
**Effect of Internet Use on Health**		
Negative	287	17.3
No effect/positive effect	1368	82.7
**Effect of Internet Use on Study**		
Negative	533	32.2
No effect/positive effect	1121	67.8
**Effect of Internet Use on Family**		
Negative	299	18.1
No effect/positive effect	1355	81.9
**Outcome Variables**		
**CESD-10 Score**	9.69	6.16
Non-depressed	807	45.6
depressed	854	54.4
**PSQI Score**	5.16	2.68
Non-sleeping disturbance	986	60.0
Sleeping disturbance	658	40.0

**Table 2:** Correlation between IAT score, PSQI score and CESD score.

Variables	IAT Score	PSQI Score	CESD Score
IAT score	1		
PSQI score	0.247	1	
	<0.0001		
CESD score	0.324	0.561	1
	<0.0001	<0.0001	

**Table 3:** Univariable associations of problematic Internet use, sleeping disturbances and depressive symptoms with socio-demographic factors and negative effects of Internet use.

Variables	Problematic Internet Use			Sleeping Disturbances			Depressive Symptoms		
	*N*	Mean	*p*-Value	*N*	Mean	*p*-Value	*N*	Mean	*p*-Value
**Gender**									
Female	801	34.99	<0.001 **	792	5.38	0.002 *	801	10.59	<0.001 **
Male	857	38.72		852	4.96		860	8.85	
**School Grade**									
7th grade	522	36.75	0.892	520	4.72	<0.001 **	522	8.82	<0.001 **
8th grade	596	37.12		592	4.94		599	9.51	
9th grade	540	36.84		532	5.84		540	10.72	
**Father’s Education Background**									
Primary school or less	476	35.12	0.016 *	476	5.87	<0.001 **	476	10.15	0.023 *
High school	767	36.48		765	4.96		767	9.67	
University or more	410	39.52		412	4.85		410	9.02	
**Mother’s Education Background**									
Primary school or less	665	36.21	0.271	663	4.91	0.259	660	8.98	0.047 *
High school	872	36.76		870	4.82		868	9.15	
University or more	120	37.49		119	5.13		120	9.46	
**Family Income**									
<CNY 20,000	477	36.06	0.237	474	5.36	0.058	480	9.72	0.180
CNY 20,000–69,999	643	36.66		638	4.98		643	9.26	
≥CNY 70,000	390	37.63		385	5.25		390	9.96	
**Most Courses in the Last Report Card**									
80–100 or A’s	753	36.02	0.035 *	747	5.21	0.206	755	9.06	<0.001 **
60–79 or C’s	494	37.16		491	4.98		494	9.99	
Below 60 or F’s	120	39.71		119	5.36		121	11.34	
**Single-Child Family**									
No	908	36.7	0.38	895	5.18	0.841	908	9.81	0.365
Yes	692	37.31		691	5.15		695	9.53	
**Intact Family**									
No	114	40.1	0.026 *	113	5.96	0.001 *	114	11.9	<0.001 **
Yes	1531	36.66		1518	5.11		1534	9.53	
**Effect of Internet Use on Health**									
Negative	287	43.85	<0.001 **	284	6.05	<0.001 **	287	12.4	<0.001 **
No effect/positive effect	1365	35.44		1354	4.98		1368	9.1	
**Effect of Internet Use on Study**									
Negative	533	42.63	<0.001 **	527	5.89	<0.001 **	533	11.99	<0.001 **
No effect/positive effect	1118	34.17		1112	4.81		1121	8.55	
**Effect of Internet Use on Family**									
Negative	299	45.47	<0.001 **	298	6.44	<0.001 **	299	13.22	<0.001 **
No effect/positive effect	1352	35.02		1341	4.88		1355	8.88	

* *p*-value less than 0.05; ** *p*-value less than 0.001.

**Table 4:** Multiple regression models for testing the relationships between problematic Internet use, sleep disturbance and depressive symptoms.

Variables	*β*	Std *β*	SE	95% CI	*p*-Value	Adj *R*^2^
Model I: Depressive symptoms mediates the relationship between problematic Internet use and sleeping disturbance						
PIU → SD	0.048	0.246	0.005	0.039, 0.057	<0.001	0.060
PIU → DS	0.146	0.323	0.010	0.125, 0.166	<0.001	0.104
DS → SD	0.232	0.536	0.009	0.214, 0.251	<0.001	0.317
PIU → SD/DS	0.014	0.072	0.004	0.006, 0.022	0.001	0.317
Sobel test, *Z* = 12.70, *p* < 0.001						
Model II: Problematic Internet use mediates the relationship between depressive symptoms and sleep disturbance						
DS → SD	0.242	0.560	0.009	0.225, 0.260	<0.001	0.313
DS → PIU	0.717	0.323	0.052	0.616, 0.818	<0.001	0.104
PIU → SD	0.014	0.072	0.004	0.006, 0.022	0.001	0.317
DS → SD/PIU	0.232	0.536	0.009	0.214, 0.251	<0.001	0.317
Sobel test, *Z* = 3.39, *p* < 0.001						

All regression equations were controlled for gender, school grade and intact family because only these variables were significantly associated with sleep disturbance in univariate analyses; Std *β,* standardized beta coefficient; Adj *R*^2^, adjusted *R*^2^; PIU, problematic Internet use; SD, sleep disturbance; DS, depressive symptoms.

## References

[1] Lenhart A., Purcell K., Smith A., Zickuhr K. Social Media and Young Adults. Pew Internet & American Life Project. http://www.pewinternet.org/Reports/2010/Social-Media-and-Young-Adults.aspx.

[2] CINIC (2014). Chinese Adolescents Internet Survey Report. http://www.cnnic.cn/hlwfzyj/hlwxzbg/qsnbg/201406/t20140611_47215.htm.

[3] Subrahmanyam K., Kraut R., Greenfield P., Gross E. (2000). The impact of home computer use on children’s activities and development. Future Child..

[4] Yuan K., Qin W., Liu Y., Tian J. (2011). Internet addiction: Neuroimaging findings. Commun. Integr. Boil..

[5] Harris S. (2011). Too Much Internet Use “Can Damage Teenagers’ Brains”. Mail Online.

[6] Wang H., Zhou X., Lu C., Wu J., Deng X., Hong L. (2011). Problematic internet use in high school students in Guangdong province, China. PLoS ONE.

[7] Wu X., Tao S., Zhang Y., Zhang S., Tao F. (2015). Low Physical Activity and High Screen Time Can Increase the Risks of Mental Health Problems and Poor Sleep Quality among Chinese College Students. PLoS ONE.

[8] Bener A., Bhugra D. (2013). Lifestyle and Depressive Risk Factors Associated with Problematic Internet Use in Adolescents in an Arabian Gulf Culture. J. Addict. Med..

[9] Durak M., ŞENOL-DURAK E. (2013). Associations of Social Anxiety and Depression with Cognitions Related to Problematic Internet Use in Youths. Egitim. Bilim..

[10] Tonioni F., D’Alessandris L., Lai C., Martinelli D., Corvino S., Vasale M., Fanella F., Aceto P., Bria P. (2012). Internet addiction: Hours spent online, behaviors and psychological symptoms. Gen. Hosp. Psychiatry.

[11] Spada M. (2013). An overview of problematic Internet use. Addict. Behav..

[12] Brunet J., Sabiston C., O’Loughlin E., Chaiton M., Low N., O’Loughlin J. (2014). Symptoms of depression are longitudinally associated with sedentary behaviors among young men but not among young women. Prev. Med..

[13] Lam L. (2014). Internet Gaming Addiction, Problematic Use of the Internet, and Sleep Problems: A Systematic Review. Curr. Psychiatry Rep..

[14] Jenaro C., Flores N., Gómez-Vela M., González-Gil F., Caballo C. (2007). Problematic internet and cell-phone use: Psychological, behavioral, and health correlates. Addict. Res. Theory.

[15] Canan F., Yildirim O., Sinani G., Ozturk O., Ustunel T., Ataoglu A. (2013). Internet addiction and sleep disturbance symptoms among Turkish high school students. Sleep Biol. Rhythms..

[16] Van den B. (2004). Television viewing, computer game playing, and Internet use and self-reported time to bed and time out of bed in secondary-school children. Sleep.

[17] Choi K., Son H., Park M., Han J., Kim K., Lee B., Gwak H. (2009). Internet overuse and excessive daytime sleepiness in adolescents. Psychiatry Clin. Neurosci..

[18] Punamäki R., Wallenius M., Nygard C., Saarni L., Rimpela A. (2007). Use of information and communication technology (ICT) and perceived health in adolescence: The role of sleeping habits and waking-time tiredness. J. Adolesc..

[19] Spear L. (2000). The adolescent brain and age-related behavioral manifestations. Neurosci. Biobehav. Rev..

[20] Manglick M., Rajaratnam S., Taffe J., Tonge B., Melvin G. (2013). Persistent sleep disturbance is associated with treatment response in adolescents with depression. Aust. N. Z. J. Psychiatry.

[21] Fernando A., Samaranayake C., Blank C., Roberts G., Arroll B. (2013). Sleep disorders among high school students in New Zealand. J. Prim. Health Care.

[22] Cheung T., Yip P. (2015). Depression, Anxiety and Symptoms of Stress among Hong Kong Nurses: A Cross-sectional Study. Int. J. Environ. Res. Public Health.

[23] Robert J., Hoffmann R., Emslie G., Rintelmann J., Moore J., Armitage R. (2005). The relationship between sleep and clinical features of depression in children and adolescents. Sleep.

[24] Lustberg L., Reynolds C. (2000). Depression and insomnia: Questions of cause and effect. Sleep Med. Rev..

[25] Johnson E., Roth T., Breslau N. (2006). The association of insomnia with anxiety disorders and depression: Exploration of the direction of risk. J. Psychiatr. Res..

[26] Ehlers C., Frank E., Kupfer D. (1988). Social Zeitgebers and Biological Rhythms. A Unified Approach to Understanding the Etiology of Depression. Arch. Gen. Psychiatry.

[27] Baglioni C., Battagliese G., Feige B. (2011). Insomnia as a predictor of depression: A meta-analytic evaluation of longitudinal epidemiological studies. J. Affect. Disord..

[28] Danielsson N., Harvey A., Macdonald S., Jansson-Frojmark M., Linton S. (2013). Sleep disturbance and depressive symptoms in adolescence: The role of catastrophic worry. J. Youth Adolesc..

[29] Fairholme C., Manber R. (2014). Safety behaviors and sleep effort predict sleep disturbance and fatigue in an outpatient sample with anxiety and depressive disorders. J. Psychosom. Res..

[30] Ford D., Kamerow D. (1988). Epidemiologic-Study of Sleep Disturbances and Psychiatric-Disorders—An Opportunity for Prevention. JAMA.

[31] Li L., Liu J., Xu H., Zhang Z. (2015). Understanding Rural-Urban Differences in Depressive Symptoms among Older Adults in China. J. Aging Health.

[32] Tan Y., Ma D., Chen Y., Cheng F., Liu X., Li L. (2015). Relationships between Sleep Behaviors and Unintentional Injury in Southern Chinese School-Aged Children: A Population-Based Study. Int. J. Environ. Res. Public Health.

[33] Xu Z., Su H., Zou Y., Chen J., Wu J., Chang W. (2012). Sleep quality of Chinese adolescents: Distribution and its associated factors. J. Paediatr. Child. Health.

[34] Lai C., Mak K., Cheng C., Watanabe H., Nomachi S., Nomachi S., Bahar N., Ho R.C., Young K. (2015). Measurement Invariance of the Internet Addiction Test Among Hong Kong, Japanese, and Malaysian Adolescents. Cyberpsychol. Behav. Soc. Netw..

[35] Baron R., Kenny D. (1986). The moderator-mediator variable distinction in social psychological research: Conceptual, strategic, and statistical considerations. J. Personal. Soc. Psychol..

[36] MacKinnon D., Lockwood C., Hoffman J., West S., Sheets V. (2002). Comparison of methods to test mediation and other intervening variable effects. Psychol. Methods.

[37] Cheung L., Wong W. (2011). The effects of insomnia and internet addiction on depression in Hong Kong Chinese adolescents: An exploratory cross-sectional analysis. J. Sleep Res..

[38] Gradisar M., Gardner G., Dohnt H. (2011). Recent worldwide sleep patterns and problems during adolescence: A review and meta-analysis of age, region, and sleep. Sleep Med..

[39] Amaral M., Pereira C., Martins D., Serpa C., Sakellarides C. (2013). Prevalence and risk factors for insomnia among portuguese adolescents. Eur. J. Pediatr..

[40] Guo J., Chen L., Wang X., Liu Y., Chui C., He H., Qu Z., Tian D. (2012). The relationship between internet addiction and depression among migrant children and left-behind children in China. Cyberpsychol. Behav. Soc. Netw..

[41] Lin S., Tsai C. (2002). Sensation seeking and internet dependence of Taiwanese high school adolescents. Comput. Human Behav..

[42] Dalbudak E., Evren C., Aldemir S., Coskun K., Ugurlu H., Yildirim F. (2013). Relationship of internet addiction severity with depression, anxiety, and alexithymia, temperament and character in university students. Cyberpsychol. Behav. Soc. Netw..

[43] Yougn B. (2004). Diffusion and usage patterns of the Internet in Korea and Japan: A comparison of policy and cultural factors. Dev. Soc..

[44] Do Y., Shin E., Bautista M., Foo K. (2013). The associations between self-reported sleep duration and adolescent health outcomes: What is the role of time spent on Internet use?. Sleep Med..

